# Functional role and evolutionary contributions of floral gland morphoanatomy in the Paleotropical genus *Acridocarpus* (Malpighiaceae)

**DOI:** 10.1371/journal.pone.0222561

**Published:** 2019-09-17

**Authors:** Isabel Reis Guesdon, André Márcio Amorim, Renata Maria Strozi Alves Meira

**Affiliations:** 1 Universidade Federal de Viçosa, UFV, Departamento de Biologia Vegetal, Viçosa, Minas Gerais, Brazil; 2 Universidade Estadual de Santa Cruz, UESC, Departamento de Ciências Biológicas, Ilhéus, Bahia, Brazil; 3 Herbário Centro de Pesquisas do Cacau, CEPEC, Itabuna, Bahia, Brazil; Instituto de Investigaciones en Recursos Naturales, Agroecologia y Desarrollo Rural (IRNAD), Universidad Nacional de Rio Negro - Sede Andina, ARGENTINA

## Abstract

The stereotyped floral morphology of Neotropical Malpighiaceae species—zygomorphic and calyx with oil glands—is correlated with oil-bee pollination. In contrast, the floral trends of the Paleotropical lineages are actinomorphy and lack of calyx glands, probably due to the absence of oil-collecting bees. The Paleotropical genus *Acridocarpus* is an exception because of its zygomorphic, gland-bearing flowers. Glands throughout *Acridocarpus* inflorescences were morphoanatomically evaluated to verify the occurrence of patterns related to species and geographic distribution. Herbarium (25 species) and fresh samples of *A*. *longifolius* were processed according to standard anatomical techniques. To verify the presence of glucose and protein, strip tests were applied to the exudate. Fresh samples were fixed and submitted to histochemical tests. Based on the occurrence, number and placement of glands, three distribution patterns were recognized on the bracteole and ten on the calyx. Bract, bracteole and sepal glands have a typical nectary structure with a palisade-like epidermis and vascularized parenchyma. Glands were classified as short-stalked, sessile or immersed. Histochemical tests performed on bract and sepal glands of *A*. *longifolius* were positive for proteins, polysaccharides and phenolic compounds, and negative for oil compounds. Glucose and protein were detected in the exudate. These results allow us to recognize the glands in *Acridocarpus* inflorescences as nectaries. This comprehensive morphoanatomical study helps to clarify the correlation between patterns of floral morphology and secretion consumers, as well as to better understand floral evolution in Malpighiaceae across their dispersal events.

## Introduction

The family Malpighiaceae comprises approximately 1300 species of trees, shrubs, vines climbing and rarely herbs, distributed in the Neo- and Paleotropics [1, 2, 3]. Most species usually have the following: 2-branched malpighiaceous trichomes; simple opposites leaves, with intra- or interpetiolar stipules; pentamerous bisexual flowers; androecium with 10 stamens; gynoecium superior, tricarpellate, 1-ovulate; and fleshy or dry fruits [4, 5].

Although the pantropical distribution of Malpighiaceae has been explained over the past by Gondwanan vicariance [1], fossil and phylogenetic evidence suggest a post-Gondwanan origin in the Neotropics [6], which is in agreement with Anderson’s American hypothesis [2]. This evidence combined with divergence time estimates indicate repeated migration events from the Neo- to the Paleotropics [6, 7]. In addition, the last phylogeny of Malpighiaceae identified nine Paleotropical clades [3], and most of them are placed within Neotropical lineages as sister groups [8].

Secretory structures are well documented in Malpighiaceae species. They include nectaries located throughout the leaf, which attract nectar consumers that may provide protection against herbivory [1], as well as glands on the calyx, which play an important role in pollinator attraction in the Neotropics, acting as oil-producing elaiophores [1, 2, 9]. Since these sepal glands are typical of the Neotropical species, they are considered a synaphomorphy for Malpighiaceae with multiple loss events [1], which support the American origin of the family [1, 2, 9, 10]. On the other hand, although morphoanatomical and exudate analyses of Paleotropical genera are scarce, Vogel [1] postulated that the sepal glands in these Paleotropical lineages of Malpighiaceae have become modified and seem to behave as nectaries [1, 2, 11, 12]. Therefore, Malpighiaceae constitute an interesting group to test hypotheses about floral evolution and to examine the maintenance of morphological traits, since they exhibit a typical oil-flower pollination syndrome in the Neotropics, and the dissociation with this syndrome is predominantly related to Paleotropical species [1, 13, 3, 8].

**Fig 1:**
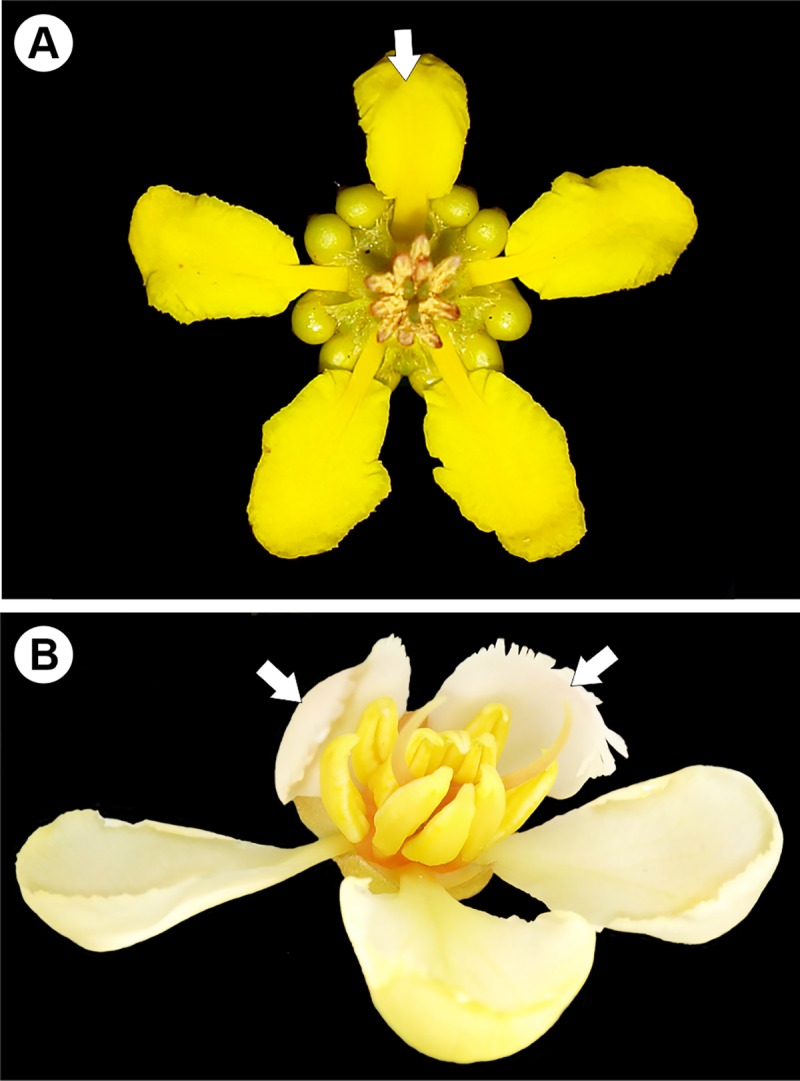
**Floral morphology of Neotropical (A) and Paleotropical Malpighiaceae (B).** (A) *Tetrapterys* sp. with one posterior petal (white arrow) and (B) *Acridocarpus longifolius*, showing two posterior petals (white arrows). Photos A and B by I. R. Guesdon.

The floral morphology of Neotropical Malpighiaceae is highly conserved and typically zygomorphic, with clawed petals, one uppermost posterior petal and calyx glands on the abaxial surface of sepals [1, 4, 5, 9, 14] (Fig 1A). The posterior petal is strongly correlated with the pollinator position to access the sepal glands [1, 2, 9, 11, 8, 15–20]. The pollinators are bees of tribes Centridini, Tapinotaspidini and Tetrapediini, which scratch their specialized legs on these glands to collect the fatty oil that is used as a larval food resource and nest coat [1, 2, 17, 18]. Interactions between oil-collecting bees and oil-flowers are a very specialized mutualism. This specialized pollination system has driven the floral evolution of Malpighiaceae in the Neotropics [2, 8], where floral traits evolved under the selective pressure of oil-bees [1, 8].

Specialist oil-collecting bees are absent in the Paleotropics [1], resulting in the loss of the selective pressure to maintain the typical Neotropical floral morphology [2]. The floral morphology of Paleotropical species presents instead actinomorphic flowers, weakly clawed petals, posterior petal absence and eglandular calyx [1, 21, 22]. The records of heteranthery, poricidal anthers and dry and dusty pollen in Paleotropical species [1, 3, 8, 20] are expected features in a pollen syndrome flower [1]. These androecium changes suggest the loss of oil-bee pollination in the majority of Paleotropical clades, and pollen is the only obvious pollinator reward [1, 3].

*Acridocarpus* Guill. & Perr. is one of the exceptional genera in the Paleotropics, as well as *Hiptage* Gaertn. and *Tristellateia* Thouars, for having some floral morphology characteristics found in Neotropical species, such as zygomorphic flowers, with posterior petals and glandular calyx [1, 11, 21, 22]. This genus comprises about 30 species distributed in Africa, including Madagascar, the Arabian Peninsula, and one species in New Caledonia [7, 8, 21, 22]. According to the phylogeny of Malpighiaceae, *Acridocarpus* composes the acridocarpoid clade with *Brachylophon* Oliv. [3], which is a monospecific genus from the Malay Peninsula and Sumatra [7]. *Acridocarpus* is characterized by the following: erect or climbing shrubs and rarely small trees, with simple and alternate leaves; yellow flowers clustered in racemes or terminal panicles, sessile pedicels with floriferous bracts and two bracteoles at the base; bracteole sometimes glandular; zygomorphic flowers; mostly with glandular calyx; weakly clawed petals, two posterior petals; poricide anthers; ovary 2-carpelate with curved styles; and fruits typically winged [1, 20, 21, 22]. Interestingly, the zygomorphy of the *Acridocarpus* flower is reoriented, with two posterior petals, two lateral petals and only one anterior petal, while the calyx presents only one posterior sepal, two lateral sepals and two anterior sepals [1, 20] (Fig 1B). The occurrence of glands on the bracteoles and calyx is commonly documented in taxonomic studies of *Acridocarpus* [21–23]. However, little attention has been given to the glands on the bracts at the base of inflorescence peduncle. The functional role and structure of these glands are not fully understood. In this work, we characterize the morphology and anatomy of these glands in *Acridocarpus* species and evaluate the gland secretion with the aim of identifying patterns across the genus, their association to species geographic distribution and their phylogenetic framework. Finally, this study contributes to clarifying patterns of floral evolution within Paleotropical Malpighiaceae.

## Materials and methods

Floral samples were obtained from herbarium material of 25 *Acridocarpus* species, from the Muséum National d’Histoire Naturelle (P), France (33 specimens in Table 1). The bracteole and sepal glands were analyzed with a stereomicroscope (Stemi 2000-C Zeiss, Gottingen, Germany) equipped with a digital camera (AxioCam ERc; Zeiss, Gottingen, Germany). The bracts at the base of inflorescence peduncle were not always well preserved and when these bracts were present, the sampling from the exsiccate was discarded, in order to avoid injuries and to preserve the herbarium collection.

**Table 1:** List of material of *Acridocarpus* from the Muséum National d'Histoire Naturelle (MNHN) used in this study, including collector and collection number.

Species	Collector and number
*Acridocarpus adenophorus* A. Juss.	Capuron 8883
*Acridocarpus alopecurus* Sprague	Haerdi 447, Sacleux 779
*Acridocarpus alternifolius* Nied.	Meikle 845, Breteler 5322
*Acridocarpus austrocaledonicus* Baill.	McPherson 3306
*Acridocarpus camerunensis* Nied.	Le Testu 7800
*Acridocarpus chevalieri* Sprague	Jaeger 3461
*Acridocarpus chloropterus* Oliv.	Schlieben 2427
*Acridocarpus congolensis* Sprague	Chevalier 28381
*Acridocarpus excelsus* A. Juss.	Schatz 2984
*Acridocarpus humbertii* Arènes	Phillipson 5989
*Acridocarpus katangensis* De Wild.	Gathy 1950
*Acridocarpus longifolius* Hook. F.	Bos 4924, Chevalier 26184
*Acridocarpus macrocalyx* Engl.	Letouzey 11775, Carvalho 3455
*Acridocarpus monodii* Arènes & Jaeger ex Birnbaum & J.Florence	Griaule 60, Birnbaum 615
*Acridocarpus natalitius* A. Juss.	Phillipson 3807
*Acridocarpus orientalis* A. Juss.	Popov 706
*Acridocarpus perrieri* Arènes	Rakotondrajaona 397
*Acridocarpus plagiopterus* Guill. & Perr.	Chevalier 14767; 20357
*Acridocarpus prasinus* Exell	Sita 3148
*Acridocarpus smeathmannii* Guill. & Perr.	Leeuwenberg 2409
*Acridocarpus socrotanus* Oliv.	Smith 204
*Acridocarpus spectabilis* (Nied.) Doorn-Hoekm.	Valenza 420, Birnbaum 751
*Acridocarpus vanderystii* R.Wilczek	Koechlin 6027, Chevalier 11097
*Acridocarpus vivy* Arènes	Schatz 4165
*Acridocarpus zanzibaricus* A. Juss.	Zhang 154

Besides the herbarium material, samples of bracts at the base of the inflorescence, peduncle, bracteoles, and calyx of *Acridocarpus longifolius* were obtained from a plant nursery, from collections at the Jardin Botanique de Meise, Belgium (*19391489*, *19700668*). They were fixed in FAA (formalin, acetic acid and 50% ethanol; 1:1:18 by volume) [24] for 48 h and then stored in 70% ethanol. The secretion exuded by all the glands on the inflorescence was submitted to glucose and protein concentration tests using urinalysis reagent strips (Insight, Acon Laboratories, San Diego, USA). A Sudan black test was also made to detect oil compounds on the surface of the glands.

Samples from herbarium material (Table 1) were subjected to a reversion process [25], dehydrated and stored in 70% ethanol. Samples of both herbarium and fixed fresh material were embedded in methacrylate (Historesin Leica; Heidelberg, Germany) following the manufacturer’s recommendations. Cross and longitudinal sections (5μm thickness) were made with an automatic rotary microtome (Leica RM2155, Deerfield, USA). The sections were stained with toluidine blue at pH 4.7 [26] and the slides were mounted in Permount (Fisher Scientific, NJ, USA).

The following histochemical tests were performed on the fixed samples (*Acridocarpus longifolius*): for total proteins, xylidine ponceau [27] and Coomassie blue [28]; for total polysaccharides, periodic acid–Schiff reagent—PAS [29]; for mucilage and pectin, ruthenium red and for starch, lugol [24]; for phenolic compounds, ferric chloride [24]; and for total lipids, Sudan red [30].

Images were taken using a light microscope (Olympus AX70TRF) equipped with a digital camera (AxioCam HRc; Zeiss, Gottingen, Germany) at the Laboratory of Plant Anatomy of the Federal University of Viçosa (UFV), Brazil. Scanning electron microscopy (SEM) analyses were conducted at the Center for Microscopy and Microanalysis (Viçosa, Brazil), with a LEO 1430VP (Zeiss, Cambridge, UK). The fixed samples were dehydrated, critically point dried using CO_2_ (CPD 030, Bal-Tec, Balzers, Liechtenstein), fixed on stubs and sputter coated with gold (SCD 050, Bal-tec, Balzers, Liechtenstein).

The morphological description was based mainly on Niedenzu [21, 22] and Anderson’s terminology [5, 14]. For anatomical descriptions, the glands were classified as **stalked**, when a short non-secretory stalk was present, and **immersed** or **sessile** when the secretory tissues were distributed above or below the level of the non-secretory epidermis surrounding the gland, respectively. The types of gland and distribution patterns were classified according to position in symmetrical plans. For bracts and bracteoles were observed the median or marginal placement, while for calyx, beyond marginal or intersepalar position, was observed the distribution on the dorsal, lateral or anterior sepals.

## Results

The number of sepal glands in sampled *Acridocarpus* species from different geographical distribution was confirmed (Table 2). The ranges of variation and the absence of glands reported in taxonomical studies were also checked (see Table 2). The glands on the bract peduncle of the inflorescence were described for the first time in the genus, and the bracteole glands in *A*. *austrocaledonicus* was recorded for the first time.

**Table 2:** Geographic distribution of *Acridocarpus* species, occurrence and number variations of glands on the peduncle bract, bracteole and calyx, in the species analyzed in this study and in taxonomical previous studies. - indicates absence and x indicates unknown data.

Geographic distribution	Species	Glands on the Peduncle Bract / Bracteole / calyx		
		(this study)	(previous studies)	
Madagascar	*A*. *adenophorus*	x / 2 / -	x / 1 / -	[21, 22, 31]
	*A*. *excelsus*	x / 2 / -	x / -; 1 / -	[21, 22, 31]
	*A*. *humbertii*	x / 2 / -	x / 1 / -	[32]
	*A*. *perrieri*	x / 2 / -	x / 1 / -	[31]
	*A*. *vivy*	x / 2 / -	x / 1 / -	[31]
Continental Africa	*A*. *alopecurus*	x / 1 / 3(5)	x / 1 / 3(5); 2–3	[21, 22, 33, 34]
	*A*. *alternifolius*	x /—/ 5	x /—/ 2–4; 2	[21, 22, 35]
	*A*. *camerunensis*	x /—/ 4	x / x / 3–4	[21, 22]
	*A*. *chevalieri*	x /—/ 3	x /—/ 2–3	[35]
	*A*. *chloropterus*	x /—/ 3	x /—/ 3; 2–3	[21, 22, 33, 36, 37]
	*A*. *congolensis*	x / 1 / 2	x / 1 / 2–3	[21, 22, 34]
	*A*. *katangensis*	x / 1 / 2(3)	x / 1 / 2; 2–3	[21, 22, 34, 37]
	*A*. *longifolius*	2 / (relictual ?) / 1	x /—/ 1	[21, 22, 34, 35]
	*A*. *macrocalyx*	x /—/ 2(4)	x / x / 2; 2–3	[21, 22, 34]
	*A*. *monodii*	x /—/ 4	x / x / 2	[38]
	*A*. *natalitius*	x /—/ 3	x /—/ 2–3; 4	[21, 22, 37]
	*A*. *plagiopterus*	x /—/ 2	x / x / 2; 3	[21, 22, 36, 39]
	*A*. *prasinus*	x / 1 / 4	x / -; 1 / 2–3	[21, 22, 33, 34]
	*A*. *smeathmanni*	x / 1 / 3	x / 1 / 3; 2 (3–4)	[21, 22, 33, 34, 36]
	*A*. *spectabilis*	x /—/ 4	x / x / 5–10	[40]
	*A*. *vanderystii*	x /—/ 5	x / 1 / 2–3	[41]
	*A*. *zanzibaricus*	x /—/ 3	x /—/ 2; 3; 2–3	[21, 22, 33, 36]
Arabian Peninsula	*A*. *orientalis*	x /—/ 5	x / x / 2–3; 1–5	[21, 22, 42]
	*A*. *socotranus*	x /—/ 2	x / x / 2	[21, 22]
New Caledonia	*A*. *austrocaledonicus*	x / 1 / -	x /—/ x	[21, 22]

### Morphology and anatomy of bract and bracteole glands

**Fig 2:**
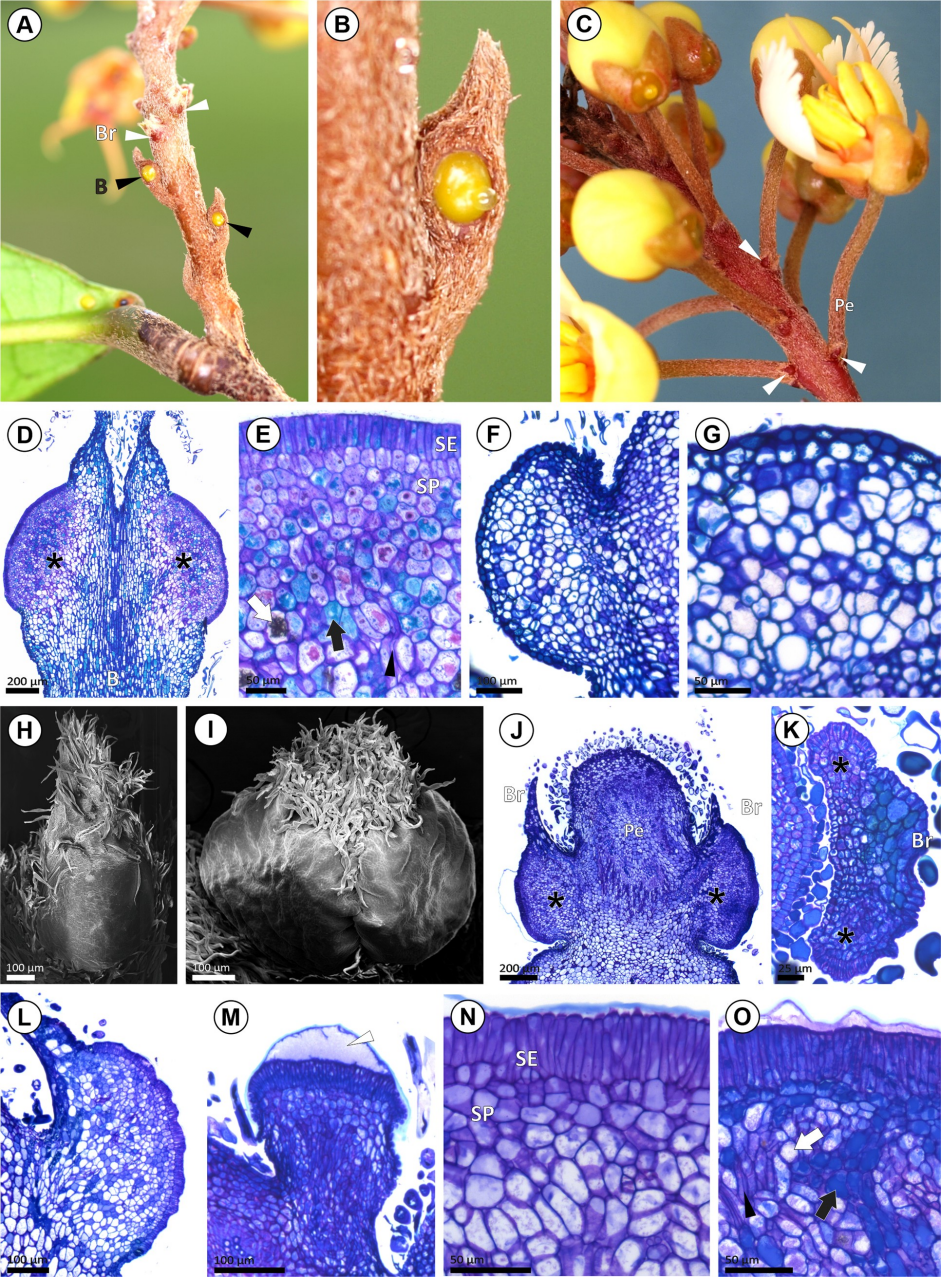
Morphoanatomy of bract and bracteole glands in *Acridocarpus*. (A-C) General view of inflorescence axis; showing glandular bract (B; black arrowheads) and bracteole (Br; white arrowheads) on the base of the pedicels. (D) Paradermal section of bract in *A*. *longifolius*, note the bract glands (asterisks). (E) Detail of the bract gland showing secretory epidermis (SE) and secretory parenchyma (SP), with voluminous nucleus and a dense-staining cytoplasm. (F, G) Bracteole gland-like protrusion in *A*. *longifolius*, note a detail showing epidermis and non-secretory parenchyma (G). (H, I) SEM image of bracteoles, one-glandular bracteole in *A*. *smeathmanni* (H) and two-glandular bracteole in *A*. *perrieri* (I). (J) Transversal section of one-glandular bracteoles (asterisks) on the pedicel (Pe) of *A*. *smeathmanni*. (K) Transversal section of two-glandular bracteole (asterisks) in *A*. *vivy*. (L) Sessile arrangement of bracteole gland in *A*. *prasinus*. (M) Stalked bracteole gland in *A*. *humbertii*, note the subcuticular space (white arrowhead). (N, O) Detail of bracteole gland in *A*. *prasinus* (N) and *A*. *humbertii* (O). Highlighting the secretory epidermis (SE) and the secretory epidermis (SP), note the presence of cystals (white arrow), phenolic idioblast (black arrow) and the vascularization (black arrowhead). Photos A-C by I. R. Guesdon.

The bract of the inflorescence peduncle was analyzed only in the fresh samples of *Acridocarpus longifolius*. However, we believe that all *Acridocarpus* species bear such bracts, which are deciduous on the mature inflorescence. On the bracts examined from the fresh samples of *Acridocarpus longifolius*, two greenish glands were observed (Fig 2A and 2B), while one reddish gland-like protrusion was recorded on the bracteoles (Fig 2A and 2C). The bract gland comprises a secretory epidermis and vascularized secretory parenchyma (Fig 2D and 2E). Although a gland-like protrusion was evident on the bracteole of *A*. *longifolius* (Fig 2A and 2C), and curiously, no secretory features were observed (Fig 2F and 2G). The shape of the bract glands and gland-like protrusion of *A*. *longifolius*, and the bracteole glands from herbarium samples, are globose with an orbicular-oblong outline (Fig 2A, 2B, 2H and 2I).

Regarding the bracteole gland position, three groups were recognized: **Group I**, one basilaminar median gland (Fig 2H, 2J and 2L), in *Acridocarpus alopecurus*, *A*. *austrocaledonicus*, *A*. *congolensis*, *A*. *katangensis*, *A*. *prasinus* and *A*. *smeathmanni*; **Group II**, two glands medially positioned (Fig 2I and 2K), in *A*. *adenophorus*, *A*. *excelsus*, *A*. *humbertii*, *A*. *perrieri*, and *A*. *vivy*; and **Group III**, eglandular, in *A*. *alternifolius*, *A*. *camerunensis*, *A*. *chevalieri*, *A*. *chloropterus*, *A*. *macrocalyx*, *A*. *monodii*, *A*. *natalitius*, *A*. *plagiopterus*, *A*. *spectabilis*, *A*. *vanderystii*, *A*. *zanzibaricus*, *A*. *orientalis*, and *A*. *socotranus*.

The anatomical constitution of the bracteole glands on samples from herbarium specimens was the same observed for the bract glands on samples from fresh material of *Acridocarpus longifolius*. The secretory epidermis cells are typically arranged in a palisade-like layer and have densely stained cytoplasm with a conspicuous nucleus and thick cuticle, which develops a subcuticular space (Fig 2M and 2O). No secretory pores or ruptured cuticles were observed (Fig 2H and 2I). The secretory parenchyma comprises a few layers of cells (Fig 2N and 2O), frequently filled with phenolic compounds (Fig 2O). Idioblasts containing druse crystals were often observed scattered in the secretory parenchyma (Fig 2O).

The majority of species have sessile bracteole glands (Fig 2J and 2L), while stalked glands (Fig 2M) were observed on the bracteoles of *Acridocarpus adenophorus*, *A*. *excelsus*, *A*. *humbertii* and *A*. *perrieri*. Stalked glands are usually associated with a flat secretory surface, while sessile bracteole glands possess a convex surface.

### Morphology and anatomy of sepal glands

**Fig 3:**
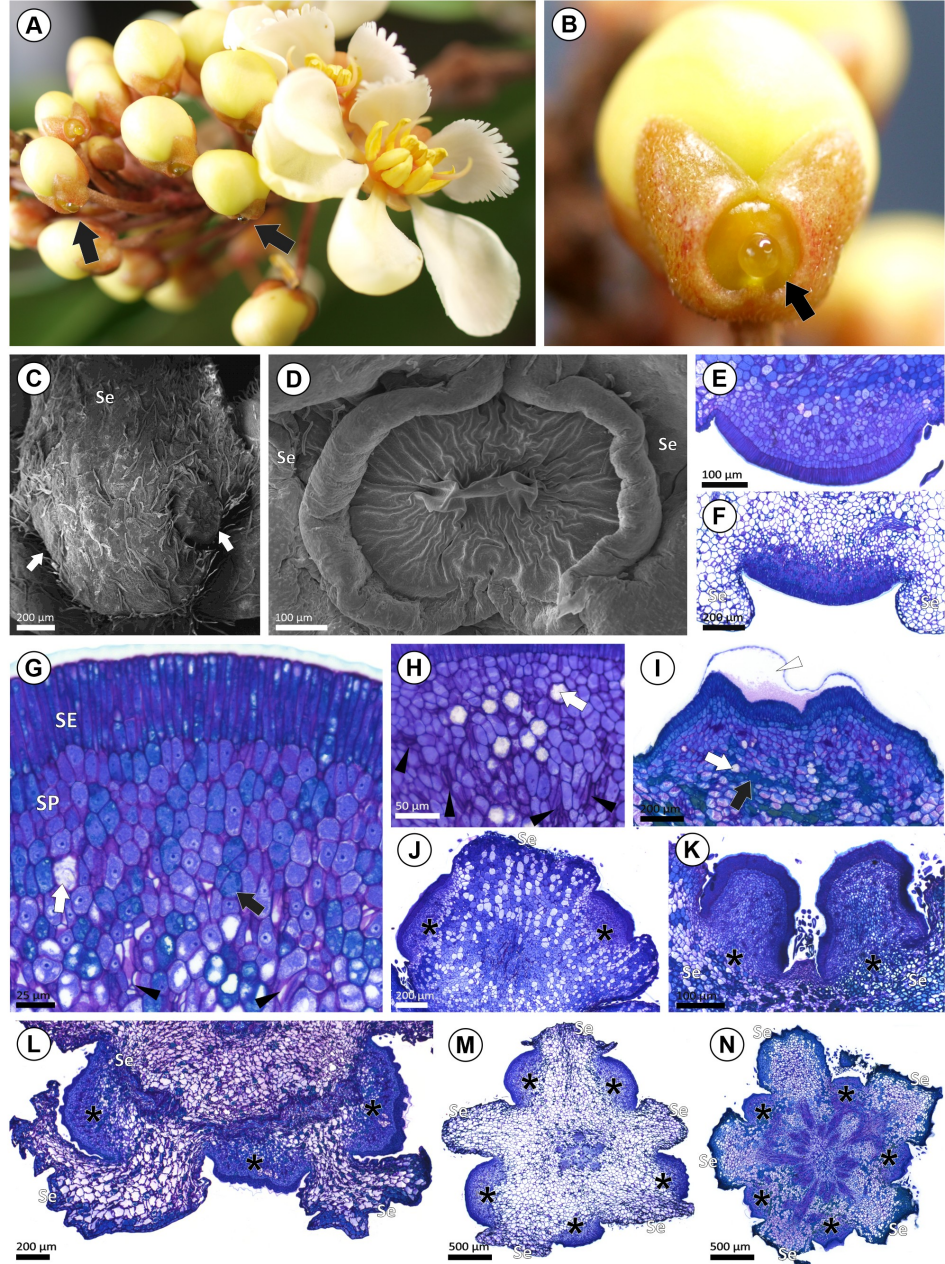
Morphoanatomy of sepal glands in *Acridocarpus*. (A, B) General view of inflorescence in *A*. *longifolius* (A) showing sepal gland of floral buds exuding a nectar drop (black arrows), the magnification is displayed in (B). (C) SEM image of marginal sepal (Se) glands (white arrows) in *A*. *smeathmanni*. (D) SEM image of the intersepalar gland of *A*. *longifolius*. (E, F) Transversal sections of a sessile sepal gland in *A*. *smeathmanni* (E) and immersed sepal gland of *A*. *longifolius* (F). (G-I) Detail of secretory tissues of sepal gland note the voluminous nucleus and a dense-staining cytoplasm of secretory epidermis (SE) and secretory parenchyma cells (SP) in *A*. *longifolius* (G), *A*. *spectabilis* (H) and *A*. *vanderystii* (I), note crystals (white arrow), phenolic idioblast (black arrow) and subcuticular space (white arrowhead). (J-N) Sepal gland distribution patterns. (J-K) Glands (asterisks) on posterior plane; glands on the posterior sepal of *A*. *socrotanus* (K), glands on posterior and lateral sepal of *A*. *prasinus*. (L) Glands (asterisks) on ventral plane, on the anterior sepals of *A*. *alopecurus*. (M, N) Glands (asterisks) on both posterior and ventral plane in *A*. *alternifolius* (M) and *A*. *vanderystii* (N). Photos A-C by I. R. Guesdon.

One yellow gland was recorded on the calyx of fresh samples of *Acridocarpus longifolius* (Fig 3A and 3B). For the herbarium specimens analyzed, the sepal glands are marginal (Fig 3C) or intersepalar (Fig 3B and 3D). These glands are globose with an oblong outline (Fig 3C) or, in *A*. *longifolius* and *A*. *zanzibaricus*, impressed with a sagittate-acute outline (Fig 3B and 3D).

All sepal glands analyzed comprise a secretory epidermis with subcuticular space and vascularized secretory parenchyma with abundant phloem, and crystalliferous and phenolic idioblasts are common (Fig 3G and 3I). The anterior gland in *Acridocarpus longifolius* (Fig 3D and 3F) and *A*. *zanzibaricus* are immersed, while in the remaining species only sessile glands were observed on the calyx (Fig 3E, 3I and 3N).

**Fig 4:**
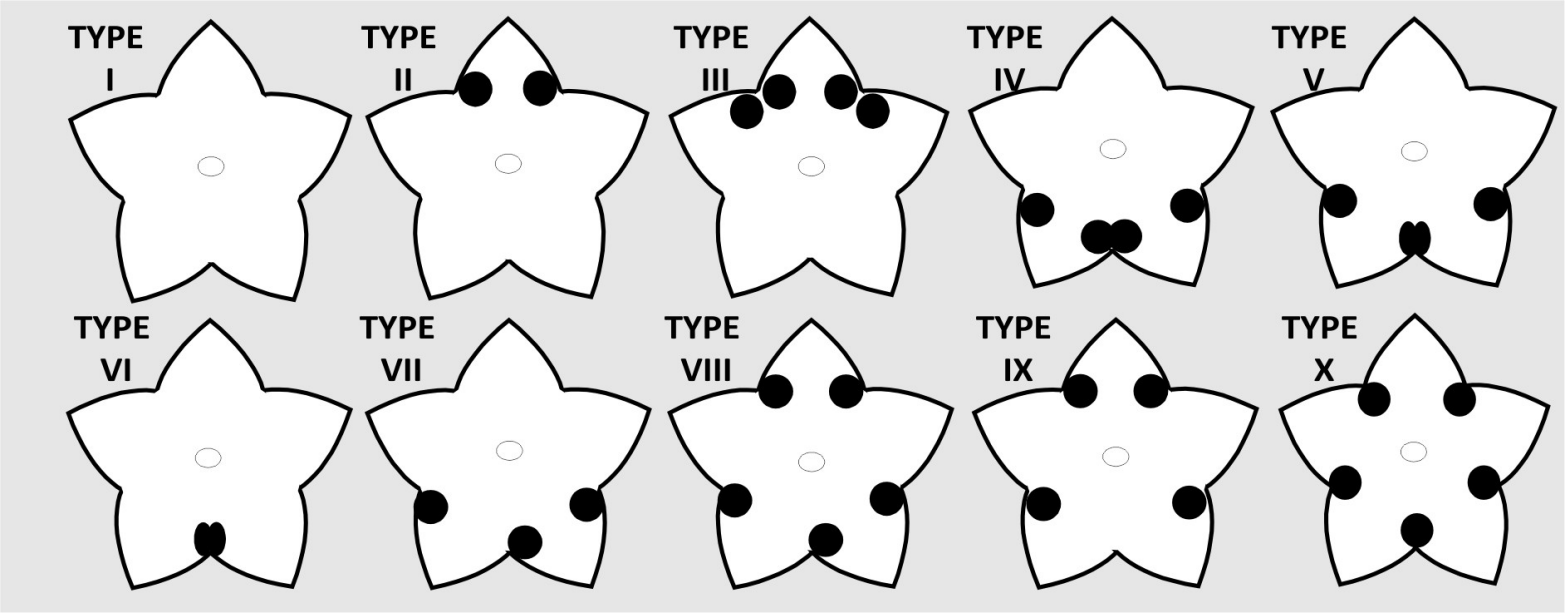
Diagram in frontal view of the morphoanatomical patterns of calyx gland distribution. **Type I,** eglandular. **Type II,** two marginal glands in posterior sepal. **Type III,** two marginal glands in posterior sepal and one marginal gland on posterior side in each lateral sepal. **Type IV,** each anterior sepal with two glands. **Type V,** distinct from type IV due to the one anterior gland in an intersepalar position. **Type VI,** one gland in the intersepalar portion of the anterior sepal pair. **Type VII,** two marginal glands in one of the anterior sepals and one marginal gland in the other anterior sepal. **Type VIII,** two marginal glands in the posterior sepal, two marginal glands in one of the anterior sepals and one marginal gland in the other anterior sepal. **Type IX,** two marginal glands in the posterior sepal and one marginal gland on the posterior side of each anterior sepal. **Type X,** one gland in each intersepalar portion.

Regarding sepal gland distribution patterns, the glands were restricted to the posterior sepal (Fig 3J and 3K), to the sepals of the ventral plane (Fig 3L) or distributed on all sepals (Fig 3M and 3N); ten morphoanatomical types were established and are illustrated in Fig 4. **Type I**: eglandular calyx, observed in in *Acridocarpus adenophorus*, *A*. *austrocaledonicus*, *A*. *excelsus*, *A*. *humbertii*, *A*. *perrieri*, and *A*. *vivy*; **Types II**: two marginal glands in posterior sepal of *A*. *congolensis*, *A*. *katangensis*, *A*. *macrocalyx*, *A*. *plagiopterus*, and *A*. *socotranus*; **Type III**: two marginal glands in posterior sepal and one marginal gland on posterior side in each lateral sepal in *A*. *katangensis*, *A*. *macrocalyx*, and *A*. *prasinus*; **Type IV**, each anterior sepal with two glands in *A*. *monodii* and *A*. *spectabilis*; **Type V**: distinct from type IV due to the one anterior gland in an intersepalar position of *A*. *zanzibaricus*; **Type VI**: one gland in the intersepalar portion of the anterior sepal pair in *A*. *longifolius*; **Type VII**: two marginal glands in one of the anterior sepals and one marginal gland in the other anterior sepal, founded in *A*. *alopecurus*, *A*. *chevalieri*, *A*. *chloropterus*, *A*. *smeathmanni*, and *A*. *natalitius*; **Type VIII:** two marginal glands in the posterior sepal, two marginal glands in one of the anterior sepals and one marginal gland in the other anterior sepal in *A*. *alopecurus*, *A*. *alternifolius*, and *A*. *orientalis*; **Type IX:** two marginal glands in the posterior sepal and one marginal gland on the posterior side of each anterior sepal in *A*. *camerunensis*, and **Type X:** one gland in each intersepalar portion in *A*. *vanderystii*.

In types II and III the glands are distributed only on the posterior plane of the calyx, while in types IV–VII they are on the sepal on the ventral plane, and in types VIII–X on both planes. However, intraspecific variations were recorded that point out different types in *A*. *alopecurus* (Types VII and VIII), and in *A*. *katangensis* and *A*. *macrocalyx* (Types II and III).

**Fig 5:**
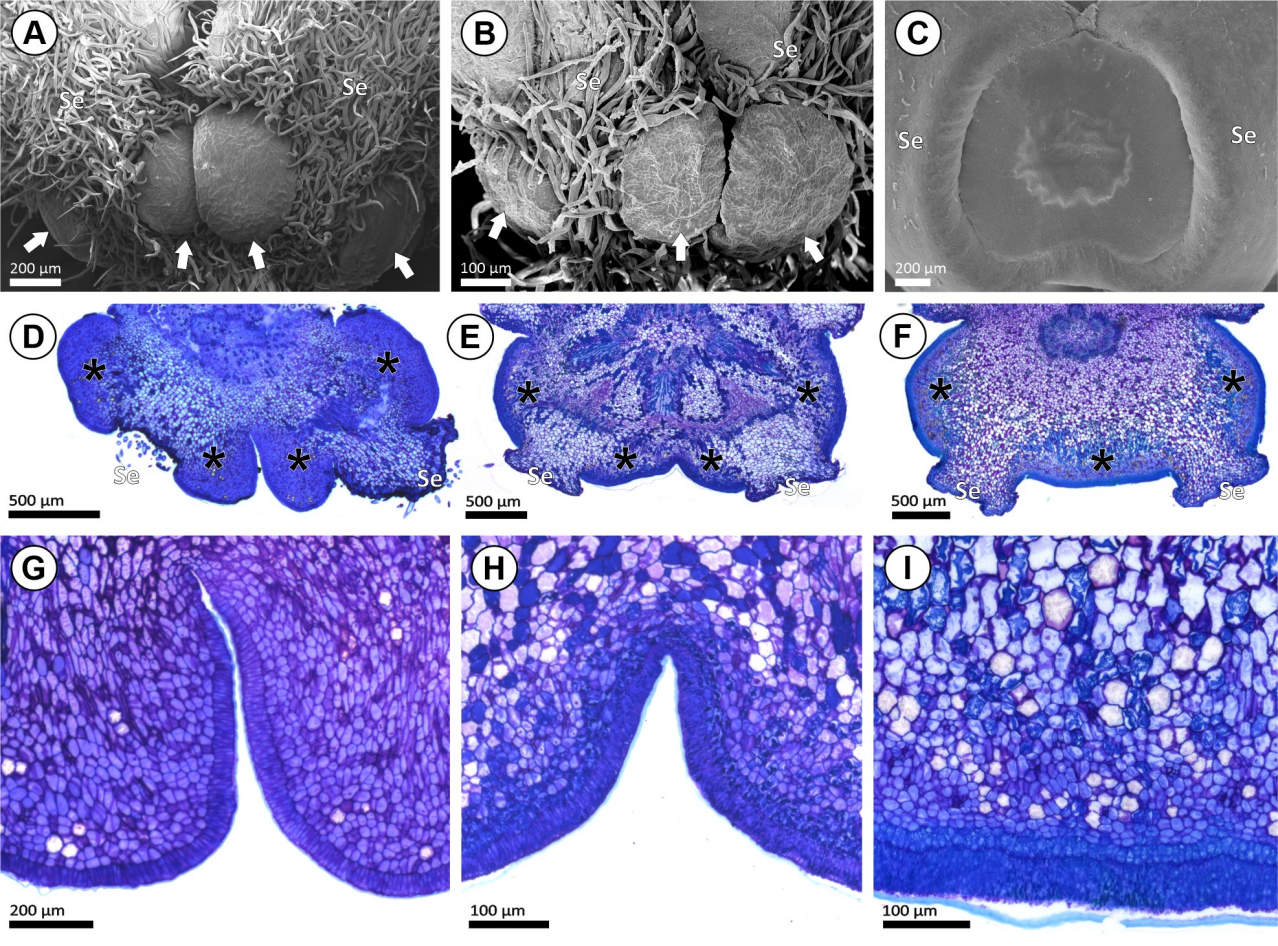
Morphoanatomy of sepal glands in *Acridocarpus* species. (A-C) SEM images of sepal glands (Se), showing two adjacent glands on the anterior sepal pair in *Acridocarpus spectabilis* (A) and *A*. *monodii* (B), and a single gland shared by the anterior sepal pair in *A*. *longifolius* (C), with a sagittate-acute outline; the white arrows are point sepal glands. (D-F) Transversal cross sections of the calyx, note the gland (asterisks) distribution in *A*. *spectabilis* (D), *A*. *monodii* (E) and *A*. *zanzibaricus* (F). (G-I) Different degrees of glandular fusion: secretory epidermis of adjacent glands juxtaposed, in *A*. *spectabilis* (G); adjacent glands sharing the epidermis and secretory parenchyma in *A*. *monodii* (H) and complete sharing of secretory tissues in *A*. *zanzibaricus* (I).

The sepal glands in most *Acridocarpus* species are small and paired at least in one sepal (Fig 4); and even for the marginal glands of adjacent sepals a non-secretory intersepalar region was observed (e.g., in *Acridocarpus prasinus*, Fig 3K). However, adjacent glands show different degrees of fusion in some cases (Fig 5A–5C). In *A*. *spectabilis*, adjacent glands of the anterior sepals are close to each other and the non-secretory intersepalar region is reduced (Fig 5D and 5G), while in *A*. *monodii* the intersepalar region between the adjacent glands is actually secretory, suggesting fusion, since they share the secretory epidermis and the secretory parenchyma (Fig 5E and 5H). Another degree of fusion was observed in *A*. *longifolius* and *A*. *zanzibaricus* (Fig 5F and 5I), which have adjacent glands that share the secretory parenchyma, vascularization and the secretory epidermis, comprising a flat and homogeneous surface (Fig 5I). One medial gland was observed in all intersepalar regions of *A*. *vanderystii* (Fig 3N); however, indications of fusion were unclear.

### Floral gland secretion in *Acridocarpus longifolius*

**Fig 6:**
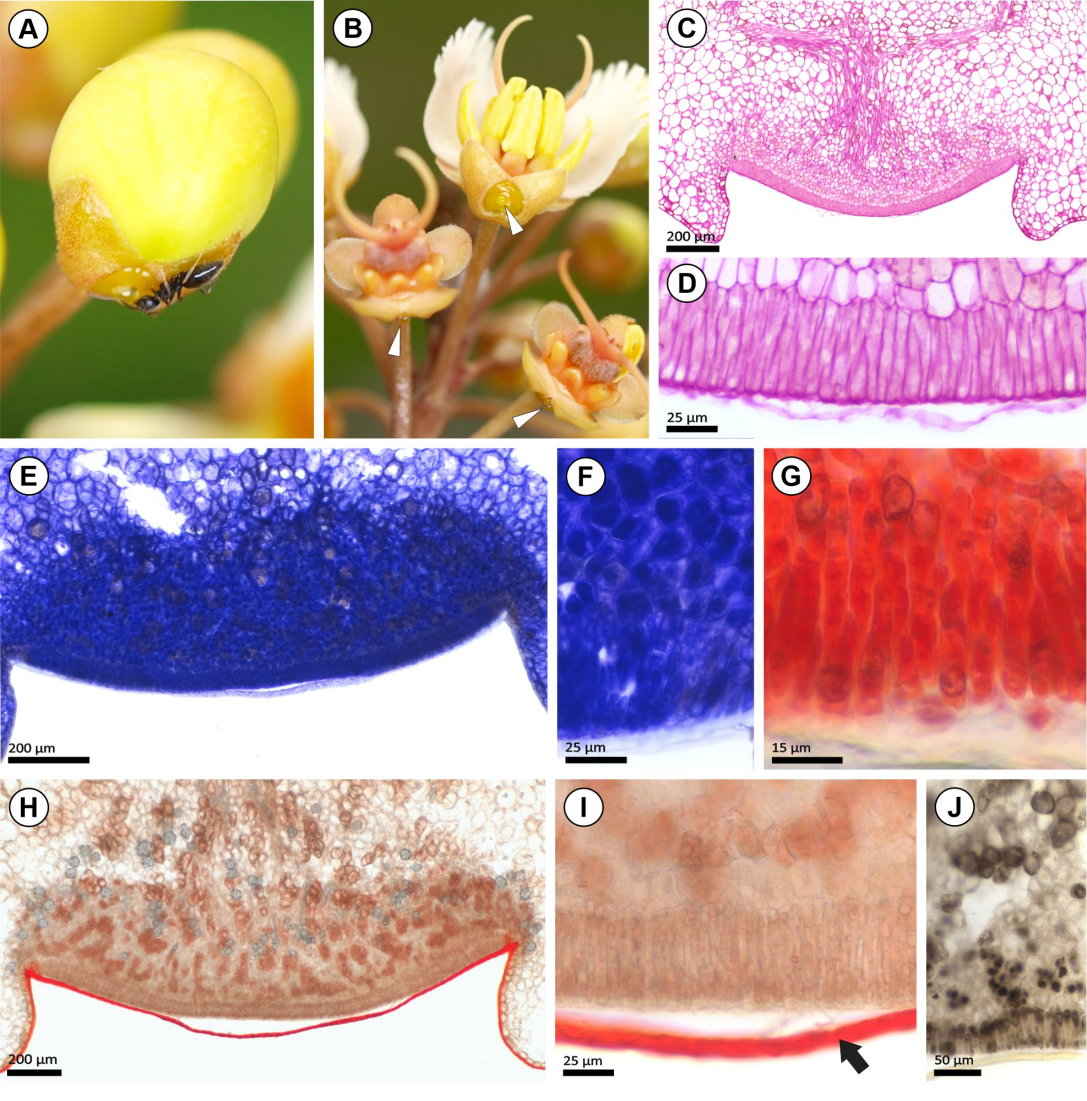
Aspects of secretion and histochemical results of sepal glands in *Acridocarpus longifolius*. (A) Nectar consumer on the sepal gland. (B) Nectar available in sepal glands of pollinated flowers and during the development of fruit (white arrowhead). (C, D) Periodic acid/Schiff reagent (PAS); magenta staining indicates neutral polysaccharides. (E-G) Protein detection by the blue stain from the Comassie blue test (E, F) and orange stain from the Xylidine Ponceau test (G). (H, I) Sudan red test; reddish color indicates total lipids, note the negative reaction for the secretory tissues (H, I) and positive reaction for the cuticle (black arrow) (I). (J) Phenolic compounds stained brown using the ferric chloride test. Photos A and B by I. R. Guesdon.

Copious secretion was recorded on the sepal glands (Fig 3A and 3B), and nectar consumers were observed visiting these structures (Fig 6A). Secretory activity begins during blooming and remains active until fruiting (Fig 6B). The bract and sepal exudate reacted with the test strips for the presence of glucose and protein. The concentration of glucose was higher in the sepal glands (1000[60] mg/dL [mmol/L]) compared to the bract glands (250[15] mg/dL [mmol/L]), while the concentration of proteins was the same in both glands (30[0.3] mg/dL[g/L]).

In agreement with the anatomical analyses of *Acridocarpus longifolius*, the histochemical test applied to the bract and sepal glands reacted positively for total polysaccharides (Fig 6C and 6D), protein (Fig 6E–6G) and phenolic compounds (Fig 6J). Proteins and phenolic compounds were distributed in the epidermis and secretory parenchyma. A positive reaction for lipid compounds was detected only on the cuticle (Fig 6H and 6I).

## Discussion

### Morphology, anatomy and composition of secretion: Functional insights

This is the first comprehensive study of morphoanatomy of *Acridocarpus* glands, and histochemical record of their secretion constitution. Based on morphoanatomy, histochemistry, persistent secretory activity, detection of glucose and protein in the exudate and the absence of lipid compounds in the exudate, we proved that the bract and sepal glands of *Acridocarpus longifolius* are actually nectaries. Vogel [43] demonstrated oil production in the elaiophores of three Neotropical species, which have been confirmed in morphoanatomical, histochemical and ultrastructural studies in several Neotropical species [44– 47]. In contrast, after the old finding of sugar in glandular secretions in two species of Paleotropical Malpighiaceae [11], studies about any glands in Paleotropical species are scarce. Until now, morphoanatomical, histochemical and ultrastructural data were restricted to only two species of *Hiptage* Gaertn. [48–50].

Although the secretion of the bracteole glands was not tested, due to their placement and similar morphoanatomy, the glands on the bracts, bracteole and sepals of *Acridocarpus* were all recognized as nectaries. The morphoanatomical characters recorded in the glands of *Acridocarpus* species analyzed have been commonly reported in nectaries of Malpighiaceae species, such as a palisade-like epidermis, with epidermal cells well juxtaposed, subcuticular space storing secretion, and idioblasts containing phenolic compounds or druse-type crystals [45, 47, 51–54].

The floral glands in the majority of investigated species of *Acridocarpus* are sessile, with the exception of stalked glands on the bracteole of *A*. *adenophorus*, *A*. *excelsus*, *A*. *humbertii* and *A*. *perrieri*. In Malpighiaceae, stalked nectaries have been recorded on the leaf and bracteole of Neotropical species [45, 51, 52] belonging to *Banisteriopis* C.B.Rob., *Lophanthera* Adr. Juss., and *Mcvaughia* W.R.Anderson [14, 45, 52]; while long stalked elaiophores were morphoanatomically described only in *Dinemandra ericoides* A. Juss., and were reported in some species-poor genera like *Dinemagonum* A. Juss., *Heladena* A. Juss. and *Henleophytum* H. Karst. [55, 22]. Immersed and embedded nectaries were previously reported, mainly in Leguminosae leaf [56, 57]; the sepal gland with secretory tissues immersed on the calyx in *A*. *longifolius*, called as “*magna*” gland by Niedenzu [22], was the first record in Malpighiaceae.

The detection of sugar and protein found in the secretions of bract and sepal nectaries in *Acridocarpus longifolius* could be related to nectar consumers, such as ants [1, 11]. This increase in nutritional richness of the nectar by the presence of protein could strengthen the ecological relations with these consumers, which prefer sugary solutions rich in amino acids [58–60]. Although few observations in the field are available to *A*. *longifolius*, the aggressive behavior of ant consumers of sepal nectar in the Paleotropical Malpighiaceae species *Hiptage benghalensis* (L.) Kurz, was associated with a protection role against inflorescence herbivory [12]. A similar interpretation is possible for *A*. *longifolius*, which the larger drops of nectar and the higher glucose concentration in their sepal glands might be involved in protecting vulnerable reproductive organs by attracting protector consumers [61, 62], since nectar composition may also vary according to plant demands [59, 63].

The presence of phenolic compounds detected in *Acridocarpus* glands, is a common feature of secretory tissues [64], and has also been reported for leaf nectaries [47, 54, 65] and elaiophores of Neotropical species of Malpighiaceae [44, 46, 47]. These compounds may provide chemical protection against herbivore attacks [63, 66].

In addition to the morphoanatomical similarity of nectaries and elaiophores reported for Neotropical species, a mixture of hydro- and lipophilic compounds detected in both secretions supports their homology [44–47, 53, 54], differing for a sugar or oil-rich secretion [1, 53]. The oil production in elaiophores of Neotropical species [11, 43], was detected in species of *Diplopterys* A. Juss. [44], *Banisteriopsis* [45], *Byrsonima* Rich. ex. Kunth and *Peixotoa* A. Juss. [46], *Galphimia* Cav. [53], *Dinemandra* A. Juss. [55], *Mcvaughia*, *Burdachia* Adr. Juss. and *Glandonia* Griseb. [47]. Unlike expected for elaiophores, the histochemical tests performed in the bract and sepal glands of *Acridocarpus longifolius*, of both field and laboratory analysis, did not detect lipids (Fig 5). Similar results were obtained for the sepal secretion of *A*. *smeathmanii* [11] and *Hiptage benghalensis* [12], where most of the compounds were reducing sugars and no lipids were found. Furthermore, the secretory activity of sepal glands in *A*. *longifolius* is premature and persistent during fruiting (Fig 5), as reported for sepal nectaries by Ren *et al*. [12]. In contrast, the elaiophore secretion starts to accumulate in the young bud stage and increases until pollinator gathering in the anthesis stage [46].

An interesting similarity to note between nectaries and elaiophores, that the secretion is accumulate in the subcuticular space [44–56]. In Neotropical species of Malpighiaceae, as a consequence of a natural process of secretion release or because of the collection behavior of oil-bees [46], the elaiophore secretion is often exposed because the cuticle rupture; this type of damage is commonly reported [44, 46, 50, 53, 55]. However, no ruptured cuticles, pores, or any damage caused by secretion consumers were observed in any glands of *Acridocarpus* analyzed, even in the herbarium material. An intact cuticle was also observed in nectaries on the bracteole of *Burdachia*, *Glandonia* and *Mcvaughia*, since the ants just slide their labrum on the gland surface [47]. Other evidence of the non-nuptial function of sepal glands in *Acridocarpus* are the weakly clawed petals, since they might make the calyx glands difficult to be accessed by pollinators, as recorded for *Hiptage benghalensis* [1]. In the latter, petals keep the calyx gland enclosed during the whole anthesis period [12]. In summary, the reoriented zygomorphic flowers in *Acridocarpus*, petal morphology, and the poricide anthers (Fig 1B) probably reflect a remarkable floral specialization to pollen-collecting pollinators, such as buzz-bees and honey bees [11]; while morphoanatomy and histochemistry of sepal glands are probably associated to plant anti-herbivore defense.

### Evolutionary trends from gland morphoanatomy and biogeography of *Acridocarpus*

The mutualistic association of Neotropical Malpighiaceae with oil-bee pollinators is evident by the floral morphology conservatism, including sepal oil-glands on the calyx. In the Neotropical species, these ten glands are typically distributed in pairs on all five sepals, but may be absent on the anterior sepal, on both anterior and lateral sepals or, rarely, the calyx is completely eglandular [1, 9, 43]. According to Vogel [1], sepal glands are absent in most Paleotropical species or, when present, vary in number (one, three or five). However, here we identified for the first time ten gland distribution patterns in the genus *Acridocarpus*.

The ventral absence of oil-glands on the calyx in the Neotropical species of Malpighiaceae has been commonly attributed to an economic reduction, due to the inability of oil-bee pollinators to use ventral glands [1]; while the eglandular calyx is associated with the oil-bee syndrome loss [2, 67–69]. Despite the lack of studies about reproductive biology in *Acridocarpus*, there is strong evidence of the loss of oil-bee syndrome, shown by the reduction/fusion or absence of sepal glands (Fig 4) and the nectar secretion. Additionally, considering the morphoanatomical evidence of homology shared by nectaries and elaiophores [1, 45, 47, 55], and the fact that nectar precedes oil secretion across the evolution [1], an expected outcome in *Acridocarpus* species, given the loss of oil-bee pollination, is the reversal to non-nuptial nectar secretion.

Although the sepal glands are taxonomically useful to distinguish Paleotropical species of *Hiptage* [70], in *Acridocarpus* these characters should be interpreted cautiously due the variability in the number of glands in the same species (Table 2), as well as in the same specimens. Intraspecific variation has been reported for some Neotropical species of Malpighiaceae, as recorded in *Byrsonima*, *Galphimia*, and *Stigmaphyllon* A. Juss., having variable numbers of glands on the anterior and lateral sepals [53, 67, 71, 72]. It is interesting to note that the high variation in number and placement of sepal glands recorded in *Acridocarpus* (Fig 4) was not described for any other Malpighiaceae genus, which may be due to a labile gene expression to determine the presence of the glands on the calyx. According to Anderson [68] this range of numerical variety is typically observed in nectaries. Additionally, Davis et al. [3, 8] proposed that the Neotropical floral morphology, including the usual elaiophore distribution in pairs on five or four sepals, are actually labile and were actively maintained by the mutualistic relation with oil-bee pollinators. Our results in *Acridocarpus* suggest that in the Paleotropics, where the species have pollen-flowers and are not under the oil-bees selective pressure, not only the secretion of sepal glands was modified to nectar, but their distribution patterns on the calyx have become highly variable (Fig 4).

Vogel [1] and Anderson [9] emphasized morphological trends of the sepal glands in Paleotropical species of Malpighiaceae, being smaller than elaiophores and typically intersepalar. Unexpectedly, in *Acridocarpus* this intersepalar placement was an exception, since a single gland in the intersepalar position was only recorded in *A*. *longifolius*, *A*. *zanzibaricus* and *A*. *vanderystii* (see Fig 4, types V, VI and X). This finding suggests that the absence of one gland of the pair on the same sepal (see Fig 4, types III, VI-IX), may be due to the numerical variability expected in nectaries. Furthermore, the occurrence of small intersepalar glands on the calyx is not exclusive to Paleotropical species, since this was reported for Neotropical species that have lost the stereotyped malpighiaceus floral morphology [68].

This intersepalar gland position was considered by Vogel [1] as the result of fusion of a pair of glands on adjacent sepals. However, Castro *et al*. [53] attribute the single gland in *Galphimia brasiliensis* A. Juss. (Malpighiaceae) to loss and not fusion of such structures. Souto and Oliveira [73] suggest that the fusion of sepal glands in *Mascagnia cordifolia* (A. Juss.) Griseb (Malpighiaceae) is based mainly on shared vascular tissue. In *Acridocarpus longifolius* and *A*. *zanzibaricus*, followed to the shared secretory tissues and vascular bundles, the sagittate-acute outline may be strong evidence of the fusion of two glands on adjacent anterior sepals (see Fig 5C for *A*. *longifolius*). The anatomy of the sepal glands of *A*. *longifolius*, *A*. *monodii*, *A*. *spectabilis* and *A*. *zanzibaricus* suggests an evolutionary sequence from juxtaposed to partially connate to completely fused glands (Fig 5E–5J), reflecting fusion of tissues from the exterior to the interior, which is in agreement with the fusion steps noted by Fahn [64].

According to phylogenetic evidence [3, 7], most Neotropical species of Malpighiaceae, like those in the byrsonimoid clade, have sepal glands, which indicates an ancestral condition [9], while their absence suggests a derived condition [73]. In the first dispersal of Neotropical ancestors to the Paleotropics, the acridocarpoid clade gave rise to Asian and African lineages (*Brachylophon* and *Acridocarpus*) [7]. Regarding that *Brachylophon* (sister group of *Acridocarpus*) is completely eglandular [22], the sepal glands in the acridocarpoid clade may be a reversal to the ancestral condition, showing morphofunctional differences from the sepal glands of most Neotropical species, probably due to the absence of oil-bee mutualism, as an adaption against herbivores.

**Fig 7:**
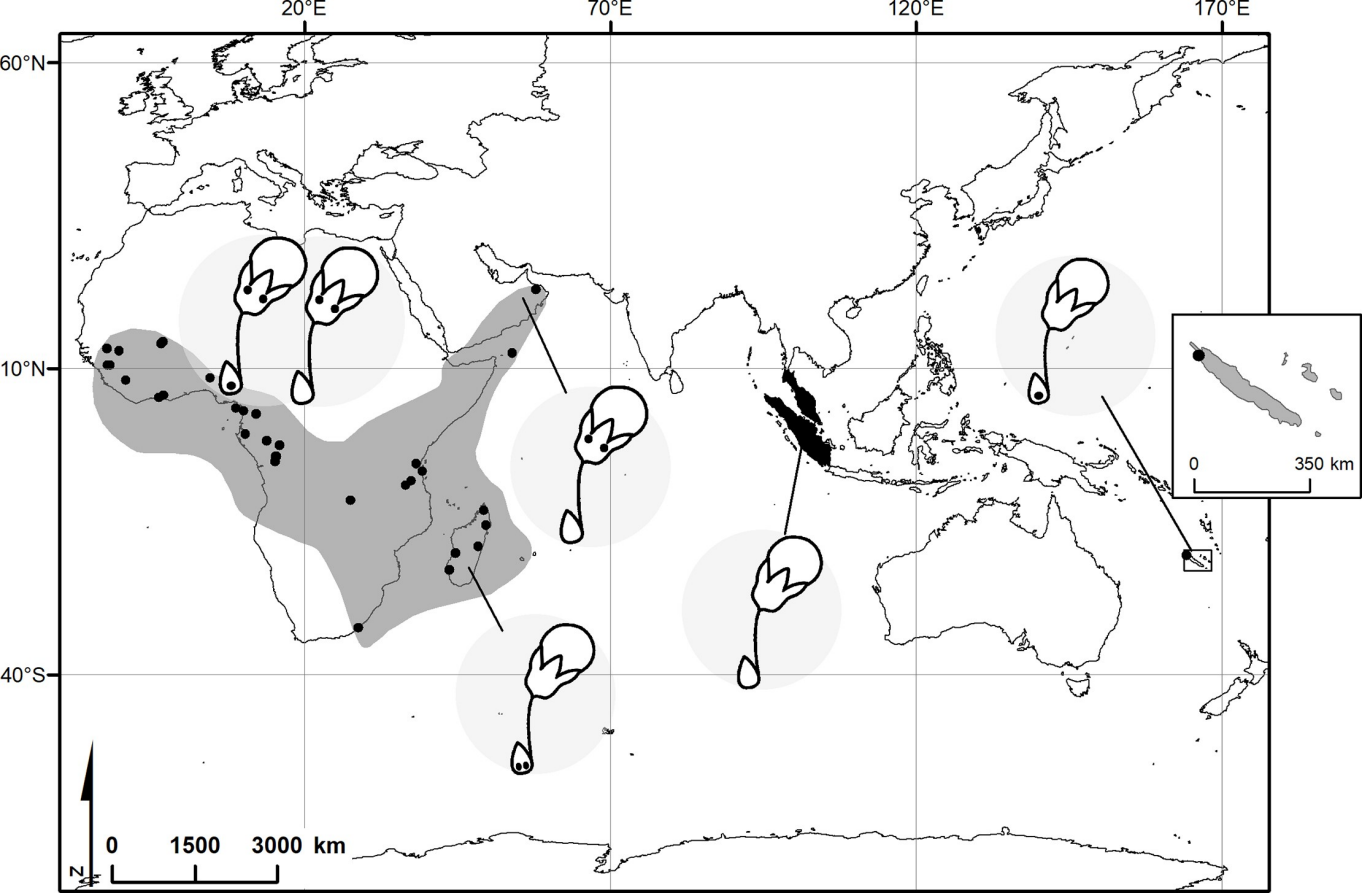
Geographic distribution and glandular condition of bracteole and calyx. *Brachylophon* (black area) in Malay Peninsula and Sumatra: eglandular. *Acridocarpus* (grey area) in Madagascar and New Caledonia: glandular bracteole and eglandular calyx; in continental Africa: eglandular bracteole (or glandular) and glandular calyx; in Arabian Peninsula: eglandular bracteole and glandular calyx. Black circles: Localities of the specimens analyzed in this study.

The reconstruction of the geographical history [7] suggest that *Acridocarpus* evolved from migrations to continental Africa, Madagascar and New Caledonia, subsequently from east to west of Africa and finally to the Arabian Peninsula. Davis *et al*. [7] proposed that *Acridocarpus* species from Madasgascar and the only species from New Caledonia are probably a disjunction by a long-distance dispersal event from Madasgascar to New Caledonia. Interesting, most Madagascar species bear stalked bracteole glands, except for *Acridocarpus vivy* that has sessile glands (Fig 2K), like in *A*. *austrocaledonicus*. *Acridocarpus longifolius* was phylogenetically placed within African species with eglandular bracteoles [7], which suggests that the gland-like protrusion of the bracteole can be a relictual signal. Additionally, an eglandular calyx associated to a glandular bracteole seems to be exclusive to *Acridocarpus* species from Madagascar and New Caledonia (Fig 7) and support the phylogenetic proximity of Madagascar-New Caledonia species demonstrated by Anderson and Davis [3]. In comparison, most species from continental Africa and Arabian Peninsula have a contrary condition, with eglandular bracteoles and glandular calyx (Fig 7).

## Conclusions

Overall, the bract, bracteole and sepal glands in *Acridocarpus* are nectaries, which is probably associated with attracting anti-herbivory protectors instead of pollinators. Due to the absence of oil-collecting bees and oil-flower mutualism in *Acridocarpus*, the different types of gland distribution patterns on the calyx identified in this study provide new evidence that such a feature is variable and labile. Our study also demonstrated the morphoanatomical similarity between nectaries and elaiophores of the Neotropical species and nectaries of the *Acridocarpus* species, which can be interpreted as a sign of their homology and the secretion transformation of sepal glands from oil to nectar, reinforcing Vogel and Anderson’s hypothesis that these structures probably evolved following a secretion change. Considering the clues pointed out by the phylogenetic framework, the glandular bracteole and eglandular calyx provide some support for the sister-taxon relationships between *Acridocarpus* species from Madagascar and New Caledonia. Finally, our findings suggest a relation between non-nuptial nectar consumers and the floral glands in *Acridocarpus*, and contribute to a better understanding of how the dispersal events from the Neo- to the Paleotropics affected the floral morphology of Malpighiaceae. Further morphoanatomical studies in other Paleotropical glandular genera may also improve the knowledge about floral evolution in Malpighiaceae.

## References

[1] Vogel S. History of the Malpighiaceae in the light of Pollination Ecology. Memoirs of the New York Botanical Garden. 1990; 55: 130–142.

[2] Anderson WR. Floral conservation in Neotropical Malpighiaceae. Biotropica. 1979; 11: 219–223.

[3] Davis CC, Anderson WR. A complete generic phylogeny of Malpighiaceae inferred from nucleotide sequence data and morphology. American Journal of Botany. 2010; 97: 2031–2048. 10.3732/ajb.1000146 21616850

[4] Anderson WR. Malpighiaceae In: Berry PE, Yatskievych K, Holst BK editors. Flora of the Venezuelan Guayana. Saint Louis: Missouri Botanical Garden; 2001 pp. 82–185.

[5] Anderson WR. Malpighiaceae In: Smith N et al editors. Flowering Plants of the Neotropics. New Jersey: Princeton; 2004 pp. 229–232.

[6] Davis CC, Bell CD, Mathews S, Donoghue MJ. Laurasian migration explains Gondwanan disjunctions: evidence from Malpighiaceae. Proceedings of the National Academy of Sciences. 2002; 99: 6833–6837. 10.1073/pnas.102175899 PMC124489 11983870

[7] Davis CC, Bell CD, Fritsch PW, Mathews S. Phylogeny of *Acridocarpus—Brachylophon* (Malpighiaceae): implications for Tertiary tropical floras and Afroasian biogeography. Evolution. 2002; 56: 2395–2405. 12583580 10.1111/j.0014-3820.2002.tb00165.x

[8] Davis CC, Schaefer H, Xi Z, Baum DA, Donoghue MJ, Harmon LJ. Long-term morphological stasis maintained by a plant–pollinator mutualism. Proceedings of the National Academy of Sciences. 2014; 111: 5914–5919. 10.1073/pnas.1403157111 PMC4000796 24706921

[9] Anderson WR. The origin of the Malpighiaceae-The evidence from morphology. Memoirs of the New York Botanical Garden.1990; 64: 210–224.

[10] Raven PH, Axelrod DI. Angiosperm biogeography and past continental movements. Annals of the Missouri Botanical Garden.1974; 61: 39–637.

[11] Lobreau-Callen D. Les Malpighiaceae et leurs pollinisateurs. Coadaptation ou coevolution. Bulletin *du Muséum* National *d'Histoire Naturelle*, *Adansonia*. 1989; 1: 79–94.

[12] Ren M, Zhong Y, Song X. Mirror-image flowers without buzz pollination in the Asian endemic *Hiptage benghalensis* (Malpighiaceae). Botanical Journal of the Linnean Society. 2013; 173: 764–774.

[13] Michener CD. The bees of the world. Baltimore: The John Hopkins University Press; 2000.

[14] Anderson WR. Malpighiaceae. The Botany of the Guayana Highland, part XI. Memoirs of the New York Botanical Garden.1981; 32: 137–147.

[15] Buchmann SL. The ecology of oil flowers and their bees. Annual Review of Ecology and Systematics. 1987; 18: 343–369.

[16] Endress PK. Diversity and evolutionary biology of tropical flowers Cambridge: University Press; 1996.

[17] Sigrist MR, Sazima M. Pollination and reproductive biology of twelve species of neotropical Malpighiaceae: stigma morphology and its implications for the breeding system. Annals of Botany. 2004; 94: 33–41. 10.1093/aob/mch108 15194562 PMC4242364

[18] Simpson BB, Neff JL. Floral rewards: alternatives to pollen and nectar. Annals of the Missouri Botanical Garden. 1981; 68: 301–322.

[19] Vinson SB, Williams HJ, Frankie GW, Shrum G. Floral lipid chemistry of *Byrsonima crassifolia* (Malpighiaceae) and a use of floral lipid by *Centris* bees (Hymenopotera: Apidae). Biotropica. 1997; 29: 76–83.

[20] Zhang W, Kramer EM, Davis CC. Floral symmetry genes and the origin and maintenance of zygomorphy in a plant-pollinator mutualism. Proceedings of the National Academy of Sciences. 2010; 107: 6388–6393. 10.1073/pnas.0910155107 PMC2851953 20363959

[21] Niedenzu F. De genere Acridocarpo. Arbeiten aus dem botanischen Institut der Staatlichen Akademie (vorm. Kgl. Lyceum hosianum). Braunsberg: Ostpreussen; 1921.

[22] Niedenzu F. Malpighiaceae In: Engler A. editor. Das Pflanzenreich. Leipzig: Verlag von Wilhelm Engelmann; 1928 pp. 1–870.

[23] Launert E. Malpighiaceae In: Edwards S. et al editor. Flora of Ethiopia and Eritrea. Uppsala: The National Herbarium, Addis Ababa and the Department of Systematic Botany; 1995 pp. 257–263.

[24] Johansen DA. Plant Microtechnique. New York: Mc Graw-Hill Book Co. Inc; 1940.

[25] Smith FH, Smith EC. Anatomy of the inferior ovary of *Darbya*. American Journal of Botany. 1942; 29: 464–471.

[26] O’Brien TP, Feder N, McCully ME. Polycromatic staining of plant cell walls by toluidina blue O. Protoplasma. 1964; 59: 368–373.

[27] Clark SG. Staining procedures. Baltimore: Williams and Wilkins; 1981.

[28] Fisher DB. Protein staining of ribboned epon sections for light microscopy. Histochemistry and Cell Biology.1968; 16: 92–96. 10.1007/BF00306214 4180491

[29] McManus JFA. Histological and histochemical uses of periodic acid. Stain Technology. 1948; 23: 99–108. 10.3109/10520294809106232 18867618

[30] Pearse AGE. Histochemistry Theoretical and Applied, 4th ed Edinburgh: Churchill Livingston; 1980.

[31] Arènes J. Malpighiacées In: Flore de Madagascar et des Comores. Catalogue of Vascular Plants of Madagascar, Tropicos.org, Missouri, Portland 1950 vol. 108: 1–176.

[32] Arènes J. Un *Acridocarpus* nouveau de Madagascar. Notulae Systematicae. 1955 ["1954"]; 15: 4–5.

[33] Launert E. Malpighiaceae Flora of tropical East Africa. British Museum (Natural History). 1968; 1–26.

[34] Wilczek R. Malpighiaceae In: Robyns W, Staner P, Demaret F, Germain R, Gilbert G, Hauman L. et al editors. Flore du Congo-Belge et du Ruanda-Urundi. Bruxelles: Institut National pour l´Etude Agronomique du Congo belge; 1958 214–234.

[35] Hutchinson J, Dalziel JM. Malpighiaceae In: Keay RWJ, Hepper FN editors. Flora of West tropical Africa, London: Crown Agents; 1958 pp. 350–354.

[36] Oliver D. Malpighiaceae In: Oliver D editor. Flora of tropical Africa. London: L. Reeve and Co; 1868 pp. 276–282.

[37] Launert E. Malpighiaceae In: Exell AW et al editors. Flora zambesiaca. London: Crow Agentes; 1963 pp. 109–225.

[38] Birnbaum P, Florence J. Validation d'*Acridocarpus monodii* Arènes & Jaeger ex Birnbaum & J. Florence, sp. nov. (Malpighiaceae). Notes sur biologie. Adansonia. 2005; 27: 235–241.

[39] Guillemin JA, Perrottet S, Richard A. Malpighiaceae *Acridocarpus*. Florae Senegambiae tentamen, Paris, vol. 29: 123–124. 1831.

[40] Doorn-Hoekman H. van. *Rhinopteryx* Niedenzu and *Acridocarpus* (G.Don) Guill. et. Perr. (Malpighiaceae) united. Acta Botanica Neerlandica. 1975; 24: 69–82.

[41] Wilczek R. Novitates africanae I (Malpighiaceae et Linaceae). Bulletin du Jardin botanique de l'état Bruxelles. 1955; 25: 303–313.

[42] Thulin M. Malpighiaceae In: Thulin M editor. Flora of Somalia. Kew: Royal Botanic Gardens; 1993 pp. 260–264

[43] Vogel S. Ölblumen und ölsammelnde Bienen. Tropische und subtropische Pflanzenwelt. 1974; 7: 283–547.

[44] Possobom CCF, Guimarães E, Machado SR. Structure and secretion mechanisms of floral glands in *Diplopterys pubipetala* (Malpighiaceae) and a Neotropical species. Flora. 2015; 211: 36–39.

[45] Araújo JS, Meira RMSA. Comparative anatomy of calyx and foliar glands of *Banisteriopsis* C. B. Rob. (Malpighiaceae). Acta Botanica Brasilica. 2016; 30: 112–123.

[46] Possobom CCF, Machado SR. Elaiophores in three Neotropical Malpighiaceae species: a comparative study. Plant Systematics and Evolution. 2017; 304: 15–32.

[47] Guesdon IR. Estruturas secretoras em linhagens Neo e Paleotropicais de Malpighiaceae: morfoanatomia, evidências funcionais e contribuições taxonômicas e evolutivas. Doctoral thesis. Universidade Federal de Viçosa. 2017.

[48] Arumugasamy K, Inamdar JA, Subramanian RB. Structure, ontogeny and secretion of oil secreting glands in *Hiptage acuminate*. Current Science. 1989; 58: 260–261.

[49] Arumugasamy K, Udaiyan K, Manian S, Sugavanam V. Ultrastructure and oil secretion in *Hiptage sericeae* Hook. Acta Societatis Botanicorum Poloniae.1993; 62: 17–20.

[50] Subramanian RB, Arumugasamy K, Inamdar JS. Studies in secretory glands of *Hiptage sericea* (Malpighiaceae). Nordic Journal of Botany. 1990; 10: 57–62.

[51] Almeida RF, Guesdon IR, Pace MR, Meira RMSA. Taxonomic revision of *Mcvaughia* W.R.Anderson (Malpighiaceae): notes on vegetative and reproductive anatomy and the description of a new species. PhytoKeys. 2019; 117: 45–72. 10.3897/phytokeys.117.32207 PMC6372574 30774506

[52] Guesdon IR, Amorim AMA, Meira RMSA. The hydrochorous Amazonian genus *Glandonia* (Malpighiaceae): New records, morphoanatomy update, and taxonomic contributions. Phytotaxa. 2018; 345: 13–25.

[53] Castro MA, Veja AS, Mulgura ME. Structure and ultrastructure of leaf and calyx glands in *Galphimia brasiliensis* (Malpighiaceae). American Journal of Botany. 2001; 88: 1935–1944. 21669626

[54] Possobom CCF, Guimarães E, Machado SR. Leaf glands act as nectaries in *Diplopterys pubipetala* (Malpighiaceae). Plant Biology. 2010; 12: 863–870. 10.1111/j.1438-8677.2009.00304.x 21040301

[55] Cocucci AA, Holgado AM, Anton AM. Estudio morfologico y anatomico de los eleoforos pedicelados de *Dinemandra ericoides*, Malpighiacea endemica del desierto de Atacama, Chile. Darwiniana. 1996; 34: 183–192.

[56] Elias TS. Foliar nectaries of unusual structure in *Leonardoxa africana* (Leguminosae) an African obligate myrmecophyte. American Journal of Botany. 1980; 67: 423–425.

[57] Gonzalez AM, Marazzi B. Extrafloral nectaries in Fabaceae: filling gaps in structural and anatomical diversity in the family. Botanical Journal of the Linnean Society. 2018; 20: 1–20.

[58] Lanza J. Response of fire ants (Formicidae: *Solenopsis invicta* and *S*. *geminata*) to artificial nectars with amino acids. Ecological Entomology. 1991; 16: 203–210.

[59] González-Teuber M, Heil M. The role of extrafloral nectar amino acids for the preferences of facultative and obligate ant mutualists. Journal of Chemical Ecology. 2009; 35:459–68. 10.1007/s10886-009-9618-4 19370376

[60] Ness JH, Morris W, Bronstein JL. For ant-protected plants, the best defense is a hungry offense. Ecology. 2009; 90: 2823–2831. 10.1890/08-1580.1 19886490

[61] Sudd JH, Sudd ME. Seasonal changes in the response of wood-ants (*Formica lugubris*) to sucrose baits. Ecological Entomology. 1985; 10: 89–97.

[62] Baker-Méio B, Marquis RJ. Context-dependent benefits from ant–plant mutualism in three sympatric varieties of *Chamaecrista desvauxii*. Journal of Ecology. 2012; 100: 242–252.

[63] Nicolson SW, Thornburg RW. Nectar chemistry In: Nicolson SW, Nepi M, Pacini E. editors. Nectaries and Nectar. Dordrecht: Springer; 2007 pp. 215–63.

[64] Fahn A. Plant Anatomy, 4 ed Oxford: Pergamon Press; 1990.

[65] Nery LA, Vieira MF, Ventrella MC. Leaf glands of *Banisteriopsis muricata* (Malpighiaceae): distribution, secretion composition, anatomy and relationship to visitor. Acta Botanica Brasilica. 2017; 31: 459–467.

[66] McKey D. The distribution of secondary compounds within plants In: Rosenthal G.A. & Janzen D.H. [eds.], Herbivores: Their Interactions with Secondary Plant Metabolites, 55–133. New York: Academic Press.; 1979.

[67] Teixeira LAG, Machado IC. Sistemas de polinização e reprodução de *Byrsonima sericea* DC (Malpighiaceae). Acta Botanica Brasilica. 2000; 14: 347–357.

[68] Anderson C. Revision of *Galphimia* (Malpighiaceae). Contribution from University Michigan Herbarium. 2007; 25: 1–82.

[69] Anderson WR, Corso S. *Psychopterys*, a new genus of Malpighiaceae from Mexico and Central America. Contribution from University Michigan Herbarium. 2007; 25: 113–135.

[70] Chen S, Funston M. Malpighiaceae. Flora of China, 2008; 11: 132–138. http://flora.huh.harvard.edu/china/mss/volume11/Malpighiaceae.pdf (Accessed 23 May. 2019).

[71] Sazima M, Sazima I. Oil-gathering bees visit flowers of eglandular morphs of the oil-producing Malpighiaceae. Botanica Acta. 1989; 102: 106–111.

[72] Carvalho PD, Borba EL, Lucchese AM. Variação no número de glândulas e produção de óleo em flores de *Stigmaphyllon paralias* A. Juss. (Malpighiaceae). Acta Botanica Brasilica. 2005; 19: 209–214.

[73] Souto LS, Oliveira DMT. Evaluation of the floral vasculature of the *Janusia*, *Mascagnia* and *Tetrapterys* species as a tool to explain the decrease of floral organs in Malpighiaceae. Flora. 2013; 208: 351–359.

